# Influence of Neighboring Plants on Shading Stress Resistance and Recovery of Eelgrass, *Zostera marina* L

**DOI:** 10.1371/journal.pone.0064064

**Published:** 2013-05-24

**Authors:** Camilla Gustafsson, Christoffer Boström

**Affiliations:** Environmental and Marine Biology, Department of Biosciences, Åbo Akademi University, Åbo, Finland; Swansea University, United Kingdom

## Abstract

Stressful environments may enhance the occurrence of facilitative interspecific interactions between plants. In several regions, *Zostera marina* occurs in mixed assemblages. However, the potential effects of plant diversity on stress responses and stability properties of *Z. marina* are poorly understood. We investigated the resistance and recovery of *Z. marina* subjected to shading (1 mo) in a field experiment lasting 2.5 mo. We shaded *Z. marina* planted in mono- and polycultures (*Potamogeton perfoliatus*, *P. pectinatus*, *P. filiformis*) in a factorial design (Shading×Richness) at 2 m depth. We estimated the resistance and recovery of *Z. marina* by measuring four response variables. Polyculture *Z. marina* lost proportionally less biomass than monocultures, thus having a greater resistance to shading. In contrast, after a 1 mo recovery period, monocultures exhibited higher biomass gain, and a faster recovery than polycultures. Our results suggest that plant species richness enhances the resistance of *Z. marina* through facilitative mechanisms, while the faster recovery in monocultures is possibly due to interspecific competition. Our results highlight the need of a much better understanding of the effects of interspecific interactions on ecosystem processes in mixed seagrass meadows, and the preservation of diverse plant assemblages to maintain ecosystem functioning.

## Introduction

Both aquatic and terrestrial research on biodiversity and ecosystem functioning suggests that diversity has positive effects on ecosystem functions such as primary production and nutrient utilization [1], [2]. In species-rich communities, the range of different species and thus, responses to environmental change or disturbance is likely higher, which affects community functioning positively [3], [4], although responses are variable and usually ecosystem- and disturbance-specific [1], [5]. Stability-diversity theories predict that community stability is enhanced through, for example, statistical averaging [4], while the relationship between population stability (individual species) and diversity is not as straightforward [6] and may for instance decrease or increase with increasing diversity [4], [5] or show no relationship [6]. Ecosystem stability is often measured in terms of stability properties such as resilience, resistance or recovery [5], [7], [8], [9]. In marine plant ecosystems resistance and recovery studies are few and demonstrated effects are generally positive [10]. For example, high functional group richness increases the recovery of macroalgae after heat stress [11], and increasing genotypic diversity may enhance the resistance and recovery of *Zostera marina* [8], [12], [13].

Positive diversity-function relationships arise due to selection and complementary mechanisms between species [14]. These same mechanisms also influence diversity-stability relationships [12], though complementary mechanisms such as facilitation may be of greater importance for population stability, i.e. stability of individuals [15]. Especially in stressful environments, positive interactions among species occur frequently [15], [16], [17], and high diversity of plant species and traits may ameliorate harsh conditions. In aquatic plant communities, plants may facilitate neighboring species by enhancing the rhizosphere oxidation during reduced light conditions, thus ameliorating anoxic sediment conditions [16], [18]. The improved oxidation can also increase the microbial process rates and the nutrient mineralization in the sediment, thereby influencing the nutrient uptake of plants both directly and indirectly [19], [20]. However, as positive and negative interactions such as competition often work simultaneously [16], [17], a shift from facilitation to competition may arise when environmental circumstances change to less stressful [21].

Seagrasses and other submerged aquatic vegetation are declining globally due to, for example, increased eutrophication, overfishing and global warming [22]. Eutrophication often results in chronic light-limitation through increased phytoplankton and macroalgal production, while dredging, sediment resuspension due to storms and sudden increases in land-runoff are stressors that result in acute light-limitation [23]. Because of lowered irradiance, the photosynthetic capacity of seagrasses is reduced causing impaired oxygen-flux from leaves to roots, subsequent root anoxia and increased sediment sulfide concentrations [24], [25], [26]. Plant physiology may also change during lowered irradiance; non-structural carbohydrate levels for instance, may decrease rapidly due to the increased carbohydrate demand to maintain growth and tissue respiration [25], [27] and nitrogen may accumulate in leaf tissue due to less growth [26], [28]. In general, seagrasses are well adapted to low light conditions and can withstand shorter periods of light deprivation through physiological and metabolic adaptations [20], [24], [29]. However, compared to many other aquatic primary producers they require higher minimum light levels to maintain a balance between oxygen production and consumption [24]. Consequently, constant light-limitation is a serious and growing problem for seagrasses occurring in areas subjected to increasing eutrophication [30].

In both temperate and tropical regions, seagrasses grow in mono- and polycultures [31], [32]. In the Baltic Sea, eelgrass grows in polycultures (commonly 5–10 angiosperms) with positive effects on ecosystem functioning and eelgrass performance [33], [34] Throughout this region, the water transparency has significantly decreased during the past 30 years, and the occurrence of both cyanobacterial and macroalgal blooms has increased [35]. Consequently, the Baltic angiosperm communities are subjected to increasing chronic light-limitation, but simultaneously grow in naturally diverse meadows where facilitative interactions potentially occur [34].

In this paper, we demonstrate for the first time, that the diversity of neighboring plants influences the performance and stability of eelgrass (*Zostera marina*) subjected to shading. Despite the general hypothesis of decreased population stability with increasing richness [4], populations of *Z. marina* have shown increasing temporal stability with increasing richness [34]. As facilitative interactions can further enhance individual species’ survival and performance during disturbance [15], we tested the hypothesis that (1) *Z. marina* growing in polycultures would have a greater resistance (measured as biomass change and carbohydrate production) to shading than plants growing in monocultures. As the recovery may also be enhanced by richness [9], [13], we further tested whether (2) the recovery of *Z. marina* growing in polycultures would be faster compared to conspecifics growing in monocultures. We also examined sediment biogeochemical conditions such as sulfide pools that may change during shading and physiological characteristics of *Z. marina* previously shown to respond to shading [26], and tested the hypothesis that (3) plants subjected to shading would show higher nutrient and sulfide accumulation and lower concentrations of soluble sugars.

## Materials and Methods

### Site Characteristics and Plant Community

The study site Fårö Island (59° 55, 219’ N, 21° 47, 711’ E) is located in the Archipelago Sea, SW Finland. Permission to conduct a field experiment at the site was granted from the private owner of the water area. The annual surface water temperature range is 0–22°C, and the salinity 5–7 psu. During the experiment the temperature ranged between 14°C in June to 20°C in August. The experimental area is semi-exposed with an average water depth of 2.0 m. The nearshore area consists of a sandy, mostly unvegetated area with sediment dominated by fine (∼ 70% 0.125 mm) and very fine (∼5% 0.0062 mm) sand with low (<0.5%) organic content [33]. At 2–5 m depth grows a seagrass meadow dominated by eelgrass *Zostera marina* (L.), perfoliate pondweed *Potamogeton perfoliatus* (L.) and sago pondweed *Potamogeton pectinatus* (L.), while stands of slender-leaved pondweed *Potamogeton filiformis* (Pers.) and horned pondweed *Zannichellia palustris* (L.) grow both within the meadow and as monospecific stands on bare sand [33], [34]. Patchy occurrences of Eurasian water-milfoil *Myriophyllum spicatum* L. and ditchgrass *Ruppia cirrhosa* (Petagna) Grande are also found within the meadow.

### Experimental Setup and Design

The experimental plant species were *Z. marina*, *P. perfoliatus*, *P. filiformis* and *P. pectinatus.* We used a replacement design [36] with a standardized initial shoot density (24 individuals) in every plot, i.e. monocultures had 24 individuals and polycultures six individuals of each species. All experimental plants were collected from nearby plant assemblages and transplanted within six hours. To facilitate *in situ* planting, plants were gently tied to 30×30 cm plastic grids (mesh size 30 mm, [33]). To investigate rhizome and root growth, *Z. marina* rhizomes were attached to the grid at the first rhizome internode. To minimize stress, plants were kept submerged during all handling. In polycultures, neighboring individuals were always heterospecific. The experimental units were planted ∼5 cm into the sediment using SCUBA.

To achieve a shading of ∼ 90% of ambient levels in the plant plots, we anchored 1 m^2^ quadratic shading screens, made of PVC pipe frames covered with a thin tarpaulin, 60 cm above the sediment surface by using rebars and small floats The following *Z. marina* (hereafter *Zm*) treatments (n = 5) were deployed: (1) *Zm* monoculture+shade, (2) *Zm* monoculture, (3) *Zm* polyculture+shade, (4) *Zm* polyculture. Plots were randomly planted in the unvegetated area between the shore and upper meadow edge in two rows with a distance of 2.5 m between plots and rows. Sparse shoots were removed two weeks prior to the experimental set-up from the area. The experiment ran from June 9– August 26, 2009, with shading screens placed over shading treatments from June 29 to July 28 (29 days). Unique plots were sampled at three occasions: (1) three weeks after establishment, hereafter Pre-shading; (2) after four weeks of shading, hereafter Post-shading; and (3) four weeks after shading screen removal, hereafter Recovery. Every sampling was destructive (n = 5 of each treatment) and 20 random plots were removed at each sampling occasion. To obtain equal sample size at each sampling event, both types of shading treatments (i.e. “shaded”, “non-shaded”) were sampled during Pre-shading, despite the shaded treatments not having received shading. Another Recovery sampling was conducted two weeks Post-shading, but since results from the two recovery samplings were redundant, we decided not to show data from the first Recovery sampling. However, data from this sampling was included in the structural equation model.

### Light Measurements

The light climate under shading screens (expressed as Photosynthetic Photon Flux Density, PPFD) was quantified at noon (12∶00–15∶00) in June when the irradiance was expected to be the highest by using an underwater quantum sensor (LI-193SA, Li-Cor, USA). The light penetration was recorded every 10 cm along a transect between screen edges. The light availability dropped rapidly 10–30 cm from the screen edge and was in the center of the screen where plants were located approximately 10% of the ambient light levels. PPFD was recorded every three hours using light loggers (HOBO Pendant® Temperature/Light Data Logger 8 K, Onset, USA) that were deployed in one non-shaded and in one shaded plot at canopy height. The loggers were calibrated using the LI-193SA. Since some fouling material settled on the loggers, they were cleansed at least twice a week. In shaded plots the light availability was approximately 13% of non-shaded plots, i.e. the light reduction was 87% compared to ambient levels. Due to the seasonality, the natural ambient light levels decreased from ∼600 µMol m^−2^ s^−1^ on June 30 to ∼400 µMol m^−2^ s^−1^ on August 25.

### Plant Growth and Biomass

To estimate the initial aboveground and belowground dry weight (dwt) plant biomass, 20–30 randomly chosen ramets of each species were sampled during the experimental start-up. Seven days prior to every sampling occasion, ten *Zm* ramets in monocultures and six ramets in polycultures were punched to determine leaf growth [37]. During each sampling, the entire plot complete with rhizomes, roots and shoots was carefully harvested, transported to the laboratory and frozen (−18°C). In the laboratory, plants were thawed, cleansed, counted, and divided into shoot, rhizome and root biomass. Material from the youngest leaves, roots and rhizomes were separated for tissue CN-determination and pooled from the same replicate (50 mg in total for each plant compartment). Plants were dried to constant weight (60°C, 48 h) and biomass (dwt) was determined with 0.01 g accuracy on an analytical scale.

### Plant Physiological Variables

To investigate the carbohydrate contents of *Zm* tissue in response to shading, a shoot with attached rhizome and roots was separated from the rest of the sampled biomass (n = 1 per replicate) and placed immediately on ice where after the material was freeze-dried. Prior to analysis, samples were ground to a fine powder and 50 mg of tissue material was retrieved for analysis. To extract soluble sugars (sucrose), samples were boiled in 90% ethanol, left to evaporate and then re-dissolved before being analyzed spectrophotometrically by using a resorcinol assay as a standard for sucrose [38].

Plant nutrient content was determined by transferring 5 mg ground material (pooled material from one replicate) to tin capsules for organic carbon (C) and nitrogen (N) elemental analysis [39]. Elemental ratios are reported as the molar C:N ratio. To investigate sulfide invasion in the plants, the total sulfur content (TS) and the stable sulfur isotopic composition (*δ* ^34^ S) in plant tissue (pooled material from one replicate) were investigated by conducting sulfur isotopic analyses. Sediment sulfides have a lower isotopic signature than seawater sulfate because of isotope fractionation by sulfate-reducing bacteria and thus, sulfur isotopic analysis may reveal whether the sulfur in plant tissue is derived from sediments, further reflecting sulfide uptake or invasion [40]. 10 mg of ground plant tissue was weighed into tin capsules, where after V_2_O_5_ was added, and the samples were analyzed by elemental analyzer combustion continuous flow isotope ratio mass spectroscopy (SerCon 20–22 IRMS) at the Stable Isotope Facility, University of California, Davis, USA. The stable isotopic signatures are reported as described in [41].

### Sediment Biogeochemistry and Water Column Nutrients

At each sampling event, sediment porewater NH_4_^+^ was collected from plots (n = 20) using rhizon soil moisture samplers (type SMS: length 100 mm, ∅ 2.5 mm), which were inserted 10 cm into the sediment and connected to 125 ml vacuum bottles. The porewater samples were kept on ice in the dark and frozen (−18°C). Water column nutrients (NH_4_^+^ and PO_4_^3−^ ) were sampled on seven occasions (30 June, 2 July, 15 July, 26 July, 5 August, 10 August and 25 August) and treated as described above. Sediment sulfide cores (depth 10 cm, ∅ 1.5 cm) were sampled at each sampling occasion from plots (n = 20). The sulfide samples were fixed and processed according to standard procedures [41], [42], [43].

### Data Analysis

Several responses variables were positively skewed and showed gamma distributions. To investigate the effects of Shading (S) and Richness (R) on response variables both gamma and normally distributed variables were analyzed with Generalized Linear Models (GENLIN in SPSS 19.0) that allow for both normally and gamma distributed variables [44]. The response variables shoot sucrose, rhizome C % and shoot C:N ratio were normally distributed but showed heterogeneous variances and were thus analyzed with a 2-way heterogeneous variance model (PROC MIXED in SAS 9.1.3). As the initial biomasses of *Z. marina* differed between mono- and polycultures, we could not compare the absolute biomasses at each sampling event; instead the relative biomass change (dwt) from the initiation of the experiment (variable x) to different sampling occasions (variable y) was calculated. The percentage values were further converted to log-ratios (log (y) – log (x) to make the values more symmetrical [34]. When comparing models belonging to different distribution families and having different link functions in Generalized Linear Models, we chose models with lower deviance values and AIC (Akaike’s Information Criteria) and better residual fit [44]. The Generalized Linear Models were based on the Maximum –Likelihood Method and the hypothesis testing on the Wald χ^2^-distribution. We further analyzed significant results with the sequential Šidák’s *post hoc* test for multiple comparisons. Variables with heterogeneous variances were tested in a 2-way heterogeneous variance model and based on lower goodness-of-fit statistics (AIC, AICC) and significantly smaller −2 Residual Log Likelihood-values, we chose the heterogeneous variance model over the original model [45]. The degrees of freedom were calculated by the Kenward-Roger method and multiple comparisons were made using the Tukey-Kramer *post hoc* test. Means and SE derived from both statistical analyses were based on estimated marginal means.

We calculated stability properties of absolute values in terms of resistance and recovery [9]. We defined resistance as the ability to withstand shading, and it was calculated as the difference in a response between the Post-shading (replicate values) and Pre-shading samplings (average of replicates). Similarly, we calculated recovery as the difference in a response between Recovery (replicate values) and Post-shading (average of replicates). Thus, recovery was defined as the change in a response after shading. We analyzed these stability measures by conducting a 1-way ANOVA with Richness as a factor. Multiple comparisons were corrected for with the sequential Šidák-method. All average values are reported ± SE.

To investigate how both shading and plant richness affected different *Zm* responses and how these responses were inter-linked; we conducted structural equation modeling (SEM) (Amos in SPSS 19.0). The structural equation model was constructed by first evaluating the theoretical effects of shading and plant richness (exogenous variables) on different measurement variables (endogenous variables). Based on both theoretical and empirical knowledge, we included pathways in the analysis that we believed would show causality. Thus, first an overall model was built from a null model with pathways added one at a time and modification indices were used to validate pathway additions and improve the model. Second, after gaining a model with high fit, it was trimmed to the most parsimonious model by removing pathways one at a time based on the χ^2^– difference test [46]. Goodness-of-fit values such as AIC and RMSEA were also compared; differences in AIC >2 between two models indicated that the models were not equivalent and the model with the lower AIC was chosen [47] while RMSEA <0.05 indicated a good fit of the model. The assumption of multivariate normality of samples was not fulfilled, thus, the samples were bootstrapped and the p-values derived were based on the Bollen-Stine bootstrap. The standardized pathway coefficients were derived from the bootstrapping.

## Results

### Environmental Variables

Water column NH_4_^+^ ranged between 0.44 µM (July) and <0.16 (August) and PO_4_^3−^between <0.02 (July) and 0.07 µM (August). The porewater NH_4_^+^ concentrations ranged between 0.67 and 127 µM with a median value of 27 µM. Immediately after shading richness affected NH_4_^+^-concentrations with lower values in polycultures, but the shading treatment had no effect (Table 1). AVS (acid volatile sulfide) pools were not affected by Richness or Shading (Table 1) and the same were true for CRS (chromium reducible sulfur) pools (data not shown).

**Table 1:** Effects of Richness and Shading on sediment biogeochemical and plant physiological responses.

		Richness (R)		Shading (S)		R×S	
		df		df		df	
		1		1		1	
		χ^2^	p	χ^2^	p	χ^2^	p
**AVS**	*Post-shading*	2.990	n.s.	2.900	n.s.	0.200	n.s.
	*Recovery*	0.280	n.s.	0.120	n.s.	0.970	n.s.
**Rhizome TS (% dwt)**	*Post-shading*	0.410	n.s.	20.890	<0.001	0.430	n.s.
	*Recovery*	0.430	n.s.	2.710	n.s.	0.520	n.s.
**Root TS (% dwt)**	*Post-shading*	4.700	n.s.	1.840	n.s.	1.240	n.s.
	*Recovery*	2.080	n.s.	0.960	n.s.	1.560	n.s.
***δ*** **^34^S_rhizome_**	*Post-shading*	7.850	0.005	16.530	<0.001	0.050	n.s.
	*Recovery*	1.600	n.s.	4.070	0.040	0.040	n.s.
**NH_4_^+^ µM**	*Post-shading*	4.340	0.038	1.530	n.s.	1.160	n.s.
	*Recovery*	0.020	n.s.	0.280	n.s.	3.740	n.s.
**Shoot N %**	*Post-shading*	0.230	n.s.	39.930	<0.001	1.480	n.s.
	*Recovery*	1.920	n.s.	0.020	n.s.	2.140	n.s.
**Root N %**	*Post-shading*	2.630	n.s.	18.920	<0.001	0.430	n.s.
	*Recovery*	0.070	n.s.	3.980	0.046	3.440	n.s.
**Shoot C %**	*Post-shading*	5.670	0.017	0.050	n.s.	6.100	0.013
	*Recovery*	1.050	n.s.	11.870	0.001	1.370	n.s.
**Shoot C:N ratio**	*Post-shading*	0.414	n.s.	34.180	<0.001	2.800	n.s.

The analyses were based on Generalized Linear Models on sediment sulfide pools (acid volatile sulfide), plant tissue sulfide isotopic signature, porewater ammonium (NH_4_^+^), plant tissue nutrients and nutrient ratios among mono- and polycultures.

### Plant Growth Variables

**Figure 1:**
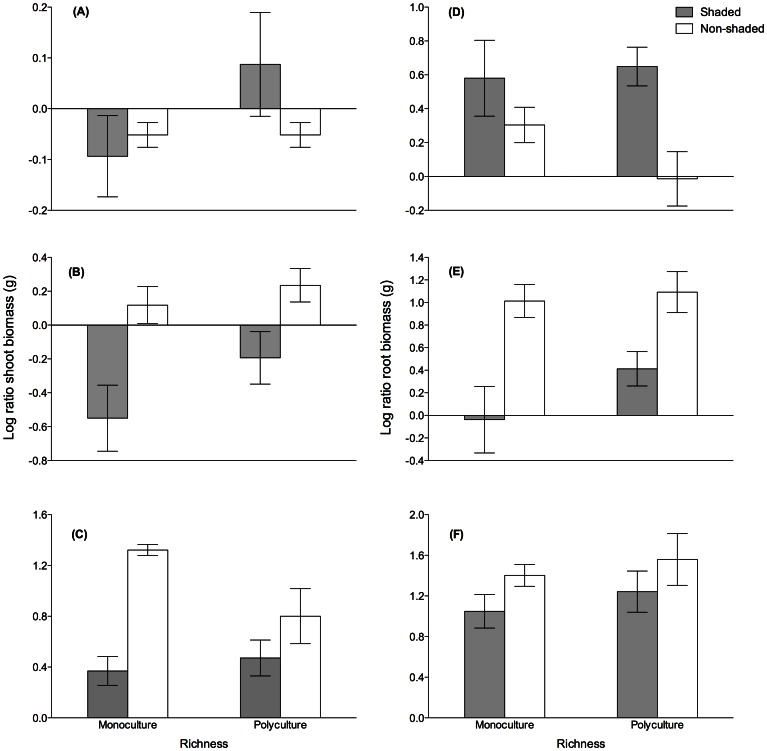
Effects of shading on biomass production. The log ratio of the change in shoot biomass (relative values) during (A) Pre-shading, (B) Post-shading and (C) Recovery and in root biomass (relative values) during (D) Pre-shading, (E) Post-shading and (F) Recovery in shaded and non-shaded mono- and polycultures. To ascertain equal samples at each sampling event, Pre-shading treatments that had not received shading were labeled shaded (see Materials and Methods). Statistical analyses presented in Table 2.

**Figure 2:**
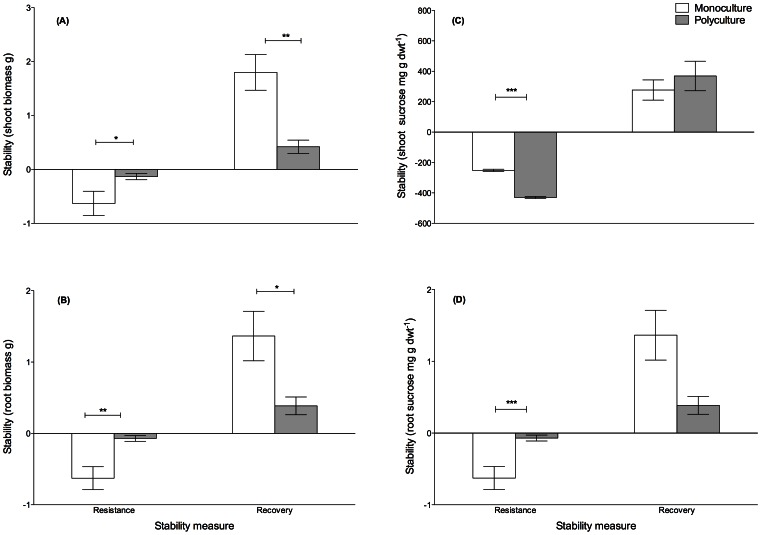
**Effects of shading on resistance and recovery of biomass production and plant tissue carbohydrate concentrations.** The stability properties resistance and recovery are calculated from the absolute values of (A) shoot biomass, (B) root biomass, (C) shoot sucrose and (D) root sucrose. Asterisks denote significant differences: * p≤0.05, ** p≤0.01, *** p≤0.001 between shaded mono- and polycultures.

Pre-shading, the changes in biomass did not differ between treatments (Fig. 1A, Table 2). Shading decreased *Zm* shoot and root biomass in both mono-and polycultures (Fig. 1B, 1E, Table 2) and these effects were still discernible after a recovery period (Fig. 1C, 1F, Table 2). Shading also decreased leaf growth but plant richness had no effect (Richness df = 1, χ^2^ = 0.31, p>0.05. Shading df = 1, χ^2^ = 13.82, p<0.001, R×S df = 1, χ^2^ = 2.18, p>0.05). Even though the change in *Zm* shoot biomass did not differ between mono- and polycultures before shading, the resistance (i.e. less difference from Post-shading to Pre-shading) of shoot biomass was significantly higher in polycultures (Fig. 2A). In turn, after 4 weeks of recovery, monocultures had reached a higher shoot biomass (Fig. 2A). Polycultures lost less root biomass to shading than monocultures (Fig 1E, Table 2) and this resulted in higher resistance among polycultures compared to monocultures, while monoculture root biomass recovered faster (Fig. 2B).

**Table 2:** Effects of Richness and Shading on plant biomass change and tissue carbohydrates.

		Richness (R)		Shading (S)		R×S	
		df		df		df	
		1		1		1	
		χ^2^	p	χ^2^	p	χ^2^	p
**Shoot biomass change**	*Pre-shading*	0.167	n.s.	1.222	n.s.	2.444	n.s.
	*Post-shading*	3.34	n.s.	17.920	<0.001	0.850	n.s.
	*Recovery*	1.52	n.s.	4.610	0.032	0.190	n.s.
**Root biomass change**	*Pre-shading*	0.930	n.s.	0.370	n.s.	0.130	n.s.
	*Post-shading*	5.197	0.015	22.050	<0.001	0.990	n.s.
	*Recovery*	0.040	n.s.	4.160	0.040	0.210	n.s.
**Root sucrose**	*Pre-shading*	11.740	0.001	8.350	0.004	0.270	n.s.
	*Post-shading*	2.750	n.s.	33.200	<0.001	0.100	n.s.
	*Recovery*	0.090	n.s.	4.750	0.030	0.220	n.s.
**Shoot sucrose**	*Recovery*	0.240	n.s.	0.180	n.s.	0.700	n.s.

Generalized Linear Models were used to analyze biomass change and plant tissue sucrose concentrations among mono- and polycultures.

### Plant Tissue Carbohydrates

**Figure 3:**
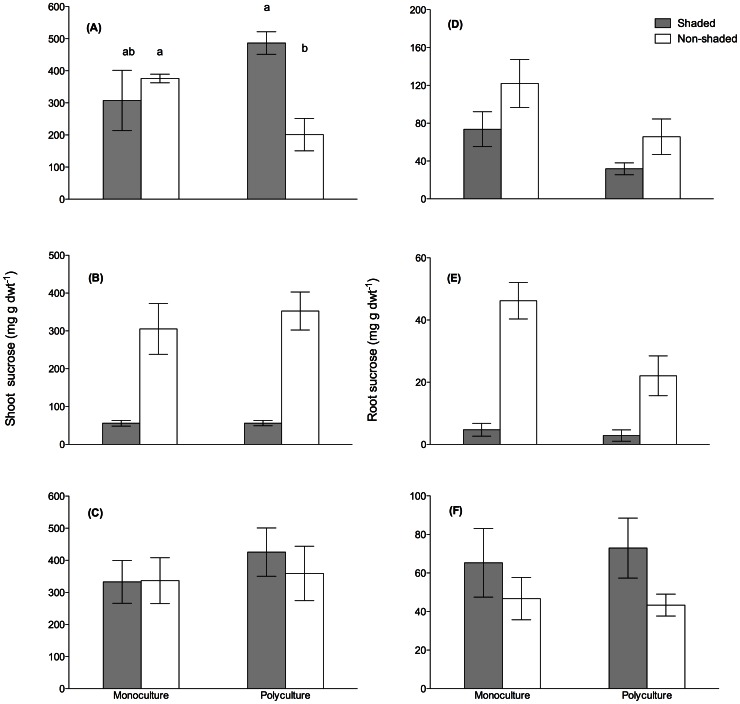
Effects of shading on plant tissue carbohydrate concentrations. Shoot sucrose concentrations during (A) Pre-shading, (B) Post-shading and (C) Recovery and root sucrose concentrations during (D) Pre-shading, (E) Post-shading and (F) Recovery of *Z. marina* grown in shaded and non-shaded mono- and polycultures. The differing letters above bars in (A) denote significant differences (p<0.05, sequential Šidák’s *post hoc* test). To ascertain equal samples at each sampling event, Pre-shading treatments that had not received shading were labeled shaded (see Materials and Methods).

Prior to shading, shoot sucrose differed between treatments (Fig. 3A, Table 3). Shading decreased the average *Zm* shoot sucrose concentrations by >80% in both mono- and polycultures, respectively, to Pre-shading levels (Fig. 3). Albeit the shoot sucrose concentration did not differ between mono- and polycultures prior to shading (Fig. 3A), the resistance to shading was lower in polycultures compared to monocultures (Fig. 2C). The shoot sucrose concentrations had recovered to non-shaded levels during Recovery (Fig. 3).

**Table 3:** Effects of Richness and Shading on tissue carbohydrates.

	df	F	P
**Shoot sucrose** *Pre-shading*			
Richness (R)	1	3.68	n.s.
Shading (S)	1	<0.001	n.s.
R×S	1	9.76	0.015
Error	7.6		
**Shoot sucrose** *Post-shading*			
Richness (R)	1	41.880	<0.001
Shading (S)	1	0.320	n.s.
R×S	1	0.310	n.s.
Error	7.65		

Shoot sucrose during Pre- and Post-shading was analyzed with a 2-way heterogeneous variance model. The degrees of freedom show ndf and ddf respectively, calculated from the Kenward-Roger method.

Prior to shading, Richness and Shading affected the root sucrose concentrations (Table 2). The concentrations dropped during shading (Fig. 3E) with the percent decrease in average sucrose concentrations being >90% in both mono- and polycultures compared to Pre-shading levels. As root sucrose concentrations differed between richness treatments prior to shading (Table 2), the difference between Post- and Pre-shading was larger among shaded monocultures compared to polycultures (Fig. 3D, 3E) and thus, the resistance significantly lower in monoculture *Zm* (Fig. 2D). After the recovery time, the shaded treatments had higher root sucrose concentrations compared to non-shaded treatments (Fig. 3F, Table 2).

### Plant Tissue Nutrients, Carbon Content and Sulfides

Both shoot C- and rhizome C-contents were lower in shaded polycultures compared to shaded monocultures (Tables 1, 4, 5A). During recovery, the rhizome C-content still differed between treatments (Table 4), but correcting for multiple comparisons removed the effect (Table 5B). After shading, shaded treatments had higher average shoot and root N-content compared to non-shaded treatments (Tables 1, 5A).

**Table 4:** Effects of Richness and Shading on plant physiological responses.

	df	F	p
**Rhizome C %** *Post-shading*			
Richness (R)	1	0.050	n.s.
Shading (S)	1	0.890	n.s.
R×S	1	12.970	0.006
Error	8.63		
**Rhizome C %** *Recovery*			
Richness (R)	1	0.52	n.s.
Shading (S)	1	0.06	n.s.
R×S	1	5.31	0.048
Error	8.65		
**Shoot C:N ratio** *Recovery*			
Richness (R)	1	0.720	n.s.
Shading (S)	1	0.730	n.s.
R×S	1	1.460	n.s.
Error	8.39		

Rhizome C % during Post-shading and Recovery and Shoot C:N ratio during Recovery were analyzed with a 2-way heterogeneous variance model. The degrees of freedom show ndf and ddf respectively, calculated from the Kenward-Roger method.

**Table 5:** Estimated marginal means ± SE of sediment sulfide pools (acid volatile sulfide), plant physiological variables and sediment NH_4_^+^ concentrations for the sampling events (**A**) Post-shading, and (**B**) Recovery.

A	Post-shading						
	Monoculture shaded		Monoculture non-shaded		Polyculture shaded		Polyculture non-shaded
AVS µmol cm_3_^−1^	0.13±0.04		0.09±0.03		0.24±0.08		0.13±0.04
Rhizome TS (% dwt)	0.35±0.02		0.25±0.02		0.34±0.04		0.23±0.01
Root TS (% dwt)	0.62±0.06		0.46±0.03		0.42±0.07		0.41±0.05
*δ* ^34^S_rhizome_	0.58±0.49		5.75±1.74		4.05±1.60		9.84±1.20
NH_4_^+^ µM	33.14±6.84		31.90±8.0		26.05±5.87		14.97±4.05
Shoot N %	1.60±0.10		1.12±0.06		1.66±0.13		0.96±0.08
Root N %	3.07±0.11		2.32±0.08		2.70±0.11		2.20±0.24
Shoot C %	36.86±0.21^a^		36.15±0.47^ab^		35.32±0.30^b^		36.18±0.21^ab^
Rhizome C %	26.96±2.04^a^		20.70±2.44^ab^		19.52±0.68^b^		25.05±0.33^a^
Shoot C:N ratio	27.62±2.17		38.18±1.84		25.50±2.0		45.69±4.45
**B**	**Recovery**						
	**Monoculture shaded**	**Monoculture non-shaded**		**Polyculture shaded**		**Polyculture non-shaded**	
AVS µmol cm_3_^−1^	0.07±0.02	0.05±0.01		0.05±0.02		0.04±0.01	
Rhizome TS (% dwt)	0.25±0.03	0.21±0.02		0.22±0.01		0.21±0.02	
Root TS (% dwt)	0.55±0.08	0.44±0.04		0.41±0.03		0.43±0.04	
*δ* ^34^S_rhizome_	2.97±1.76	6.25±0.83		5.14±1.82		7.84±1.32	
NH_4_^+^ µM	37.69±4.31	21.06±6.84		23.61±6.94		32.92±4.50	
Shoot N %	1.00±0.02	1.16±0.15		1.35±0.11		1.15±0.16	
Root N %	2.05±0.12	2.35±0.11		2.22±0.07		2.23±0.03	
Shoot C %	35.98±0.39	36.79±0.29		35.20±0.24		36.85±0.47	
Rhizome C %	20.93±2.67	26.13±1.88		21.75±0.87		24.47±0.60	
Root C %	37.77±2.47	41.46±1.25		40.52±0.20		40.52±0.28	
Shoot C:N ratio	41.90±0.66	40.07±5.07		31.46±2.39		41.84±7.07	

Results from the post-hoc test were derived from Generalized Linear Models (post hoc sequential Šidák) and 2-way heterogeneous variance models (post hoc Tukey-Kramer). Differing letters after values denote significant (p<0.05) differences.

Post-shading, the shoot C:N-ratio differed between shaded and non-shaded treatments and showed lower average ratios among shaded treatments (Tables 1, 5A). However, after the recovery period, no effects of shading could be discerned (Table 4). This indicates that a possible nitrogen accumulation in shoots during shading had normalized to non-shaded levels after 4 weeks (Table 5B). After the recovery period, root C:N ratios in both shaded and non-shaded treatments ranged between 20–21 and did not vary between diversity treatments (Richness df = 1, χ^2^ = 0.28, p>0.05, Shading df = 1 χ^2^ = 1.13, p>0.05, R×S df = 1, χ^2^ = 0.51, p>0.05).

The rhizome TS content was affected by shading (Table 1), but the root TS content was unchanged. The *δ* ^34^S_rhizome_ differed between shaded and non-shaded treatments and it also varied significantly between diversity treatments (Table 1). During Post-shading, the lowest average *δ* ^34^S_rhizome_ was found in shaded monoculture Zm (Table 5A).

### SE Model on the Interactive Effects of Shading

**Figure 4:**
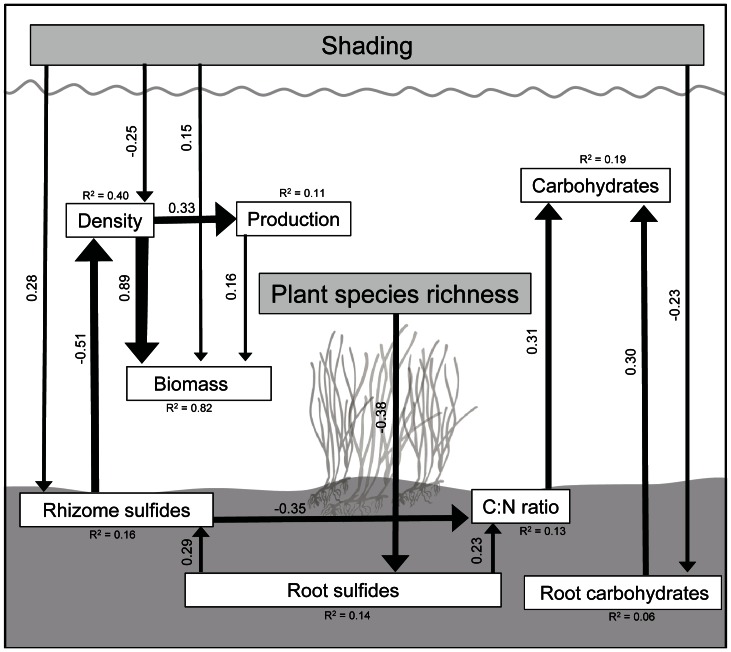
Structural equation model on overall effects of shading and plant richness on ***Zostera marina*** **.** The model fit was estimated through bootstrapping (N = 80, Bollen-Stripe bootstrap p = 0.48). The numbers next to arrows denote standardized path coefficients and all shown pathways are significantly different from 0 (p<0.05 level). The thickness of an arrow describes the strength of a correlation. If not mentioned otherwise, all response variables describe shoot parameters. Error terms are not presented graphically.

Shading affected several response variables both directly and indirectly, while plant richness only had a direct effect on one variable, namely root sulfide concentrations (Fig. 4). Shoot density and root carbohydrates were negatively affected by shading. Shading also had a positive effect on the rhizome TS content and thus indirectly affected several variables such as the shoot density and C:N ratio, while the root TS content was only affected negatively by plant richness. However, plant species richness also had an indirect effect on the rhizome TS content and the C:N ratio through the root TS content. Shoot density was strongly correlated with shoot biomass and also affected the shoot production positively (Fig. 4). Root carbohydrates affected the shoot carbohydrate content positively but shading only indirectly affected shoot carbohydrates through root carbohydrates and the C:N ratio.

## Discussion

This is the first study showing the effects of neighboring plant diversity on the population stability of the foundation species eelgrass, *Z. marina* during and after shading. We discovered that the resistance of eelgrass to shading was generally greater in polycultures. In contrast, the recovery of eelgrass was faster in monocultures. Thus, plant richness likely ameliorated effects caused by shading, which lead to higher resistance, while the occurrence of interspecific competition in polycultures may have lead to slower recovery compared to monocultures. Our results also showed that shading negatively affected eelgrass growth and carbohydrate content in such that sucrose was quickly depleted in plant tissue during shading. However, the carbohydrate production recovered quickly and four weeks after shading the carbohydrate levels were back to non-shaded levels indicating that *Z. marina* recovered fast from reduced light conditions. Thus, these results show that eelgrass can withstand serious light reductions (∼ 90%) lasting several weeks.

### Biomass Responses to Shading

Shading had clear detrimental effects on eelgrass shoot and root biomass leading to negative or less biomass change in shaded treatments compared to non-shaded treatments. The leaf production also decreased, and this is often one of the earliest responses to light reduction [25]. During shading, plant respiration likely exceeded the oxygen production leading to a use of carbohydrate reserves and thus, to sustain leaf growth, stored sucrose was used up [24], [25]. At some point however, sucrose concentrations dropped to levels that were insufficient to maintain leaf growth, which in turn declined [27]. Surprisingly, our SE model revealed a positive, but weak pathway between shading and shoot biomass, which may have arisen due to plants allocating their resources to shoot biomass in order to maintain the amount of photosynthetic tissue [48]. Nevertheless, shading primarily affected shoot biomass through the direct and indirect (rhizome sulfides) negative effects on shoot density. The robust correlation found between shoot density and biomass was not surprising as eelgrass populations often show strong shoot density-biomass relationships [49].

The non-photosynthetic tissue (roots, rhizomes) is a major respiratory part of seagrasses and during light limitation the respiratory demand increases [24]. Inhibited photosynthesis decreases the oxygen flux to roots, rhizomes and the sediment, which can turn anoxic [26]. In our study, the significant positive relationship between shading and rhizome sulfide content indicate that sulfide intrusion had occurred, possibly resulting in the rhizomes becoming anoxic. Rather than only being anoxia-induced, the negative root biomass change in shaded monocultures can also be explained by plants allocating their resources to leaf growth and maintaining the photosynthetic tissue [49] on the expense of belowground biomass. Thus, the observed root death was likely due to synergistic effects of both anoxia and sucrose depletion [25].

### Changes in Carbohydrate, Nutrient and Sulfide Concentrations

Prior to shading, shoot sucrose concentration was high [29] but dropped radically in response to shading. This can be interpreted as an increased demand of carbohydrates to maintain growth and tissue respiration. The sucrose depletion in plant tissues is a common response to reduced light among different seagrass species [27], [50]. Especially belowground compartments become totally depleted of sucrose due to the inhibited translocation of photosynthetic products from leaves to the rhizome/roots [51]. Our SE model revealed that shading affected the root carbohydrate content negatively, although the explained variance was low indicating that other factors not included in the model were of more importance in explaining variance among root carbohydrates than shading. The positive effect of root carbohydrates on shoot carbohydrates further suggests that changes in root sucrose reserves are reflected on shoot carbohydrates. As the C:N ratios were lower in shaded plants, it could suggest nitrogen accumulation occurred [26], [50] because of less nitrogen use associated with limited growth rates [28], but also due to decreasing contents of non-structural carbohydrates [50]. The sulfide isotope values in rhizomes were significantly lower in shaded plants compared to non-shaded plants but they were still in the range of values found in the northern Baltic Sea [52]. This indicates that the bacterial sulfide production in shaded plots may have been enhanced but the sulfide invasion from the sediment into tissue was nevertheless, low. However, in combination with lowered carbohydrate availability, even minor sulfide intrusion may have stressed the plants to a certain threshold where after plants started losing biomass and dying. In our SE model, the strong negative impact of especially rhizome sulfides on shoot density also supports this explanation.

### Resistance and Recovery

Facilitative mechanisms are important in diverse communities [14] and especially in harsh environments, facilitation may be a key mechanism underlying population stability [15], [16], [17]. Our results show that even though polyculture eelgrass did not lose less shoot biomass during shading compared to monocultures, its stress resistance was higher. This can be explained by improved oxygenation of the rhizosphere by neighboring plants [18] as both *Potamogeton perfoliatus* and *P. pectinatus* are more efficient oxygen releasers than eelgrass [19], [53]. The negative effect of plant species richness on root sulfides and the difference in *δ* ^34^S_rhizome_ between diversity treatments Post-shading indicates that the rhizosphere oxygenation was improved and that the sulfide production was lower in polycultures compared to monocultures. As root anoxia also affects eelgrass physiological processes negatively by for example blocking the sucrose transport from shoots to rhizomes and roots [24], improved oxygenation can have positive effects on eelgrass physiology by reducing anoxic conditions and thus, enabling sucrose translocation. Post-shading, our shaded polycultures had low carbon content in shoots and rhizomes compared to shaded monocultures. Possibly, the enhanced rhizosphere oxygenation in polycultures enabled plants to continue transporting sucrose from leaf and rhizome stores to roots, eventually leading to low carbon content in these compartments. Nevertheless, we are aware of the possibility that the lower intraspecific competition in polycultures compared to monocultures (replacement design) may to some extent have lead to enhanced resistance among *Zm* in shaded polycultures, although positive effects among species are generally believed to be caused by less interspecific competition in polycultures compared to the intraspecific competition in monocultures [54]. Furthermore, reduced intraspecific competition in polycultures does not seem to significantly enhance *Zm* biomass production (biomass change) compared to monocultures [34].

Interestingly, monoculture eelgrass increased its biomass faster after shading, leading to faster recovery. A possible cause to the found pattern may have been resource availability. Gustafsson & Boström [34] hypothesized that plants complement their resource use during stable non-stressful conditions, but the change from disturbance to recovery may have resulted in a shift from facilitation to competition in resource use [21]. Interspecific competition in polycultures can have led to increasing nitrogen limitation during recovery when plants rapidly increased their growth and biomass and, for example, *P. perfoliatus*, has a higher nitrogen demand than eelgrass [55]. The nutrient competition in polycultures may have caused a lower recovery of biomass compared to monocultures. Furthermore, the difference in porewater NH_4_^+^-concentrations between diversity treatments Post-shading, may support this explanation. In general however, the sediment NH_4_^+^-concentrations in all treatments were in the lower range of average global values (86 µM, [24], see Table 5 in comparison) and shoot nitrogen levels were below values thought to indicate nitrogen limitation (1.8% [56] see Table 5 in comparison) in both mono-and polycultures. Thus, nitrogen limitation likely affected all plants. Eelgrass can translocate nitrogen from older tissue to physiologically active tissues (e.g. young leaves) [57], but the shoot C:N ratios (measured from the youngest leaves) were much higher than in, for example, root tissue, thus, the nitrogen translocation mechanism was apparently insufficient during recovery. Therefore, we speculate that because of the rapid recovery of growth and biomass production, the nitrogen translocation was maybe not fast enough to meet the increasing demand in shoots, which resulted in nitrogen limitation. The recovery period (late summer) also coincided with naturally low nitrogen content in the water column (<16 µM). Another possible explanation to the slower recovery of polyculture plants could be due to the competition of other resources than nutrients. As some of the plant species used in the experiment are canopy forming with high primary production rates (*P. perfoliatus*, *P. pectinatus*, see [31] they may have responded to the increasing light during recovery by gaining shoot biomass rapidly. Thus, shading by other plant species may have caused the slower recovery of *Z. marina* in polycultures.

To conclude, we show for the first time that eelgrass growing in diverse plant communities has a greater resistance to shading, but not recovery. Thus, our results support earlier findings of diversity enhancing resistance to a disturbance but not the recovery. These patterns possibly arose from interspecific facilitation that increased the survival, but in contrast, when the shading ended and the community started to recover, a shift from facilitation to interspecific competition may have occurred. With increasing anthropogenic threats, eelgrass survival in areas characterized by diverse angiosperm plant communities is enhanced by the occurrence of species-rich plant assemblages. To better understand and preserve diverse plant assemblages, our results clearly highlight the need of more experimental work in both temperate and tropical mixed seagrass meadows.
